# Thermal Conductivity of Wurtzite Zinc-Oxide from First-Principles Lattice Dynamics – a Comparative Study with Gallium Nitride

**DOI:** 10.1038/srep22504

**Published:** 2016-03-01

**Authors:** Xufei Wu, Jonghoon Lee, Vikas Varshney, Jennifer L. Wohlwend, Ajit K. Roy, Tengfei Luo

**Affiliations:** 1 Aerospace and Mechanical Engineering, University of Notre Dame, Notre Dame, IN 46530; 2 Materials and Manufacturing Directorate, Air Force Research Laboratory, Wright-Patterson Air Force Base, OH 45433; 3 Universal Technology Corporation, Dayton, OH, 45342; 4 Center for Sustainable Energy at Notre Dame, Notre Dame, IN 46530.

## Abstract

Wurtzite Zinc-Oxide (*w*-ZnO) is a wide bandgap semiconductor that holds promise in power electronics applications, where heat dissipation is of critical importance. However, large discrepancies exist in the literature on the thermal conductivity of *w*-ZnO. In this paper, we determine the thermal conductivity of *w*-ZnO using first-principles lattice dynamics and compare it to that of wurtzite Gallium-Nitride (*w*-GaN) – another important wide bandgap semiconductor with the same crystal structure and similar atomic masses as *w*-ZnO. However, the thermal conductivity values show large differences (400 W/mK of *w*-GaN vs. 50 W/mK of *w*-ZnO at room temperature). It is found that the much lower thermal conductivity of ZnO originates from the smaller phonon group velocities, larger three-phonon scattering phase space and larger anharmonicity. Compared to *w*-GaN, *w*-ZnO has a smaller frequency gap in phonon dispersion, which is responsible for the stronger anharmonic phonon scattering, and the weaker interatomic bonds in *w*-ZnO leads to smaller phonon group velocities. The thermal conductivity of *w*-ZnO also shows strong size effect with nano-sized grains or structures. The results from this work help identify the cause of large discrepancies in *w*-ZnO thermal conductivity and will provide in-depth understanding of phonon dynamics for the design of *w*-ZnO-based electronics.

Wurtzite Zinc-Oxide (*w*-ZnO) is an important wide bandgap (3.4 eV)^1^ semiconductor that holds promising potential in optoelectronics and power electronics applications^2^,^3^,^4^. For those applications, especially in high power amplification devices, self-heating, which leads to hot spots with local temperatures many-folds higher than the average device temperature, can be a hindering factor limiting the device performance and reliability. Besides working as an active device material, *w*-ZnO, which is available in large bulk crystal form, has also been attractive as a substrate material for the growth of high quality epitaxial wurtzite Gallium-Nitride (*w*-GaN) layers because of the same crystal structure and the similarity in lattice constants (<2% difference)^5^,^6^. One of the crucial properties necessary for device reliability in such applications is the high thermal conductivity in order to better dissipate the unavoidable local heat^2^,^3^. Although multiple studies report thermal conductivity of crystalline *w*-ZnO, the results lack quantitative agreement between reported values, which spread over a large range (37–147 W/mK)^3^, leading to an ambiguity in the understanding of the thermal transport of *w*-ZnO and uncertainty in the device design.

Experimentally, thermal conductivity of *w*-ZnO has been measured primarily using two different methods: scanning thermal microscopy (SThM) and laser flash method. Using SThM, Florescu *et al.*^7^ measured the thermal conductivity of bulk *n*-type ZnO grown by a vapor-phase transport method in the [0001] direction and found values ranging from 98–116 W/mK at room temperature. Later, Özgür *et al.*^8^ used the same technique to measure the thermal conductivity of bulk ZnO after different thermal treatments, and reported values ranging from 46–147 W/mK at room temperature.

Compared to SThM, the laser flash method has measured much lower values of thermal conductivity. Using the latter methodology, Tsubota *et al.*^9^ found the room temperature thermal conductivity of fully sintered (at 1400 ^o^C) ZnO powder to be 49 W/mK. Although phonon transport in compacted powder is expected to be dominated by interface scattering, the thermal conductivity of those sintered samples showed a distinct 1/T temperature dependence, which indicates transport behavior is dominated by anharmonic phonon scattering– a feature generally seen in perfect or large grain crystals. Hence, it is very likely that sintering could have promoted the formation of polycrystalline ZnO with large sized grains. Another laser flash experiment on sintered ZnO powder by Olorunyolemi *et al.*^10^ showed thermal conductivity of 37 W/mK at room temperature and confirmed the 1/T trend. Katsuyama *et al.*^11^ used laser flash to measure the thermal conductivity of sintered Zn_1-x_Al_x_O alloy powder. Their reported value for the low alloying limit (x = 0.0025) was 37 W/mK at room temperature, with thermal conductivity also decreasing with increasing temperature. Barrado *et al.*^12^ used a similar method to find the thermal conductivity of a pure ZnO crystal to be 47 W/mK at room temperature. Throughout the literature, it can be generally conjectured that SThM measurements yielded higher thermal conductivities of ~100 W/mK, while laser flash measurements provided lower values (~37–47 W/mK). The large discrepancy between reported thermal conductivities of ZnO requires a theoretical reference, from which possible causes of deviation can be further analyzed.

In order to address the above issue, we use first-principles lattice dynamics, which does not require any parameterization, to predict the thermal conductivity of *w*-ZnO. This method has successfully predicted thermal conductivities of various crystals with great accuracy^13^,^14^,^15^,^16^,^17^,^18^,^19^,^20^,^21^,^22^,^23^,^24^. To determine phonon properties, the harmonic force constants are calculated from density functional perturbation theory (DFPT) while the cubic force constants are derived using the finite difference method from a set of force-displacement data obtained from density functional theory (DFT) calculations^25^,^26^. Phonon group velocity and heat capacity are calculated based on the phonon dispersion relation for each single mode. Using the cubic force constants, the phonon scattering processes are evaluated using Fermi’s Golden rule, and the thermal conductivity is calculated using the iterative solution of the Boltzmann transport equation. A more detailed description of the calculation is included in the Methods section.

We found the thermal conductivity values of pure *w*-ZnO to be 44 W/mK and 62 W/mK along the [1000] and [0001] directions, respectively. When compared to experimental literature, these results agree better with those measured using laser flash methods^9^,^10^,^11^,^12^. In studying the phonon properties of *w*-ZnO, we have made a detailed comparison to *w*-GaN – another wide bandgap semiconductor whose intrinsic thermal conductivity was found to be very high (~400 W/mK at room temperature)^13^ and share many similarities with *w*-ZnO in terms of crystal structure, lattice constants and atomic masses. A detailed comparison of relevant properties is presented in Table 1. Such a comparative study enables a detailed understanding of the root cause of the relatively low thermal conductivity of *w*-ZnO compared to *w*-GaN. In the following text, we remove ‘*w*-’ prefix and use ZnO and GaN directly to refer *w*-ZnO and *w*-GaN for convenience.

## Results and Discussion

### Thermal Conductivity Prediction and Comparison to Experiments

**Figure 1:**
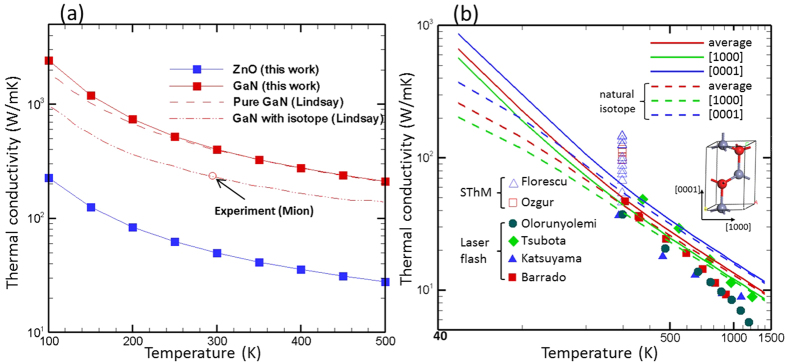
(**a**) Thermal conductivity of ZnO and GaN as a function of temperature. Lindsay – Ref. 13; Mion – Ref. 27. (**b**) Calculated ZnO thermal conductivity along different crystal directions and comparison with experimental data. Florescu – Ref. 7; Ozgur – Ref. 8; Olorunyolemi – Ref. 10; Tsubota – Ref. 9; Katsuyama – Ref. 11; Barrado – Ref. 12.

Thermal conductivity values and trends calculated using first-principles lattice dynamics (see Methods section) are shown in Fig. 1 as a function of temperature for both ZnO and GaN. As can be seen, our predicted values for isotopically enriched GaN agree very well with data from Ref. [13]. The small discrepancy at low temperatures likely originated from the *q*-space grid-size used in the calculations. At low temperatures, phonon relaxation times and thus mean free paths (MFP) become significantly larger, especially for long wavelength phonons (near Brillouin Zone center). This requires a denser *q*-space grid to achieve high accuracy. Nevertheless, the discrepancy is less than 5%. It is worth noting that the predicted thermal conductivity of pure GaN is notably higher than the experimental value (230 W/mK)^27^ at room temperature. It was found that such a large difference is rooted from isotope scattering^13^, including this led to a better agreement between calculation and experimental value (dash-dotted line in Fig. 1a).

Figure 1b shows the anisotropic thermal conductivity of ZnO along the [1000] and [0001] directions. The thermal conductivity along the [0001] direction (*i.e.*, along *c*-axis as shown in the inset) is ~40% higher than that along the [1000] direction. Figure 1b also shows the experimental thermal conductivities from different reported measurements. It is seen that values from laser flash experiments^9^,^10^,^11^,^12^ agree well with our calculations at room temperature and above. It should be noted that our predictions are on a perfect crystal and thus represent the upper limit of the ZnO thermal conductivity values. They are thus expected to be higher than experimental measurements obtained from samples where defect scattering from grain boundaries and isotopes cannot be avoided. We calculated the isotope scattering effect by incorporating the Tamura’s formula^28^ for naturally occurring isotope concentrations (Zn^64^: 48.6%, Zn^66^: 27.9%, Zn^67^: 4.1%, Zn^68^: 18.8%, Zn^70^: 0.6%; O^16^: 99.76%, O^17^: 0.038%, O^18^: 0.2%). On incorporating the natural isotope scattering effect, we found that the thermal conductivity was reduced by 12% at room temperature, unlike GaN which suffers a reduction of 40%. It is worth mentioning that the isotope scattering leads to thermal conductivity lower than some of the laser flash measurement data (two green diamond symbols at temperatures below 600 K in Fig. 1b). However, our recent study has shown that the Tamura’s formula with Matthiessen’s rule tends to overestimate the isotope scattering effect and thus underestimate thermal conductivity^29^. Actually, the experimental data are mostly smaller than the predicted thermal conductivity even after including isotope scattering. This is likely because other scattering, such as defect scattering, also exists in the experimental sample, which is not included in our calculation. In this context, we deem that the agreement between our calculations and experimental results is favorable.

On the other hand, given the variance in the experimental values from SThM measurement data, it is extremely difficult to compare the experimental and lattice dynamics results as one-to-one comparison. Nevertheless, it is not very likely that the ZnO thermal conductivity can be as high as ~100 W/mK, as reported for a few investigated samples^7^,^8^. It is possible that either the SThM experiments measured the thermal conductivity of some special phase or compounds formed due to the thermal treatment at the surfaces of samples or the accuracy of the measurement was influenced by the surface topology signal which is known to convolute with the thermal signals and lead to large uncertainties^30^,^31^,^32^.

We should also point out that the deviation between predicted and experimentally measured thermal conductivity values becomes larger at higher temperatures (lower-right corner of Fig. 1b). This is likely because higher order (>3^rd^ order) phonon scattering processes come into consideration when the temperature is very high, which are not included in our calculations, leading to an overestimation of the thermal conductivity at such high temperatures.

Overall, we conclude that the values (37–49 W/mK at room temperature) measured using laser flash for ZnO thermal conductivity are more reasonable, considering that defect and grain boundary scattering can lower the thermal conductivity. The grain sizes of the samples measured in Refs. [9, 10, 11, 12] were not clear, but they should be relatively large since the thermal conductivities scale well 1/T relation at room and high temperature –a feature seen in pure crystals or crystals with large grains.

Also, as can be seen from Fig. 1a, the thermal conductivity of ZnO is much smaller than those of GaN at all temperatures. At room temperature, the ZnO thermal conductivity, which is estimated to be 50 W/mK (averaging over all directions), is approximately eight-folds smaller than GaN. We have made detailed analysis of the phonon properties to understand this large difference in the following sections.

### Phonon properties

**Figure 2:**
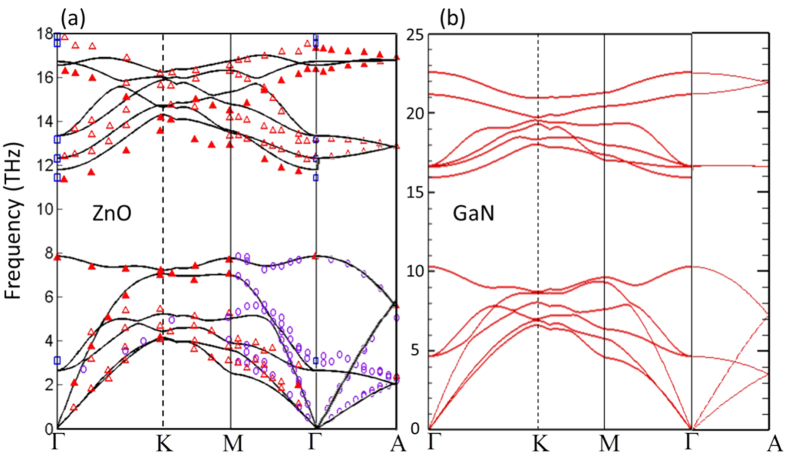
Phonon dispersion relation of (**a**) ZnO and (**b**) GaN calculated from first-principles lattice dynamics. (Symbols in panel (**a**) are experimental data: solid and open red triangles – Ref. 54; purple circles – Ref. 55, 56; blue squares – Ref. 57).

To understand the mechanism of the lower thermal conductivity of ZnO compared to GaN, we analyzed different phonon-associated properties such as phonon dispersion, group velocities, phonon relaxation times, and phonon scattering phase space. First, the calculated ZnO phonon dispersion relation shown in Fig. 2a agrees well with experimental data, although the optical phonon frequencies are slightly over-predicted. However, when we tuned the primitive cell sizes to precisely match the experimental frequencies, the variation in calculated thermal conductivity was observed to be <2%. The calculated phonon dispersion of GaN (Fig. 2b) also agrees well with experimental values^33^ and another calculation using first-principle lattice dynamics^13^ (data not shown).

**Figure 3:**
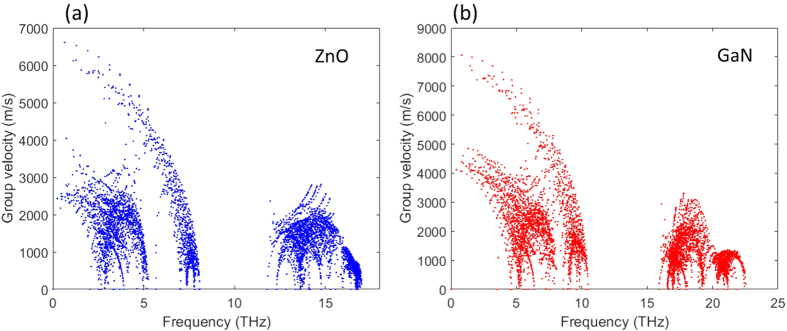
Phonon group velocities as a function of frequency for ZnO and GaN.

We then analyze the effect of heat capacity, phonon group velocity and relaxation time on thermal conductivity. According to the kinetic theory, thermal conductivity, *κ*, can be expressed as 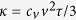, where *c*_*v*_, *v*, and *τ* respectively denote the volumetric heat capacity, the speed of sound, and the phonon relaxation time. Although the thermal conductivity calculation in this work is much more detailed and sophisticated, the kinetic theory formula offers a first order approximation for an easy comparison of thermal conductivity between ZnO and GaN. First, as seen from Table 1, there is only a slight difference in *c*_*v*_, and thus it cannot be responsible for the observed large thermal conductivity difference. The speed of sound, *v*, of GaN is about 1.3–1.5 times larger than the corresponding value for ZnO. Macroscopically, the difference in *v* can also be implied from the elastic constants (Table 1) by noting that *v* roughly equals to 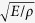, where *E* is the elastic constant of the material and 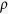 is density. From a microscopic point of view, the elastic constants are related to the interatomic bond stiffness, which is reflected as the largest harmonic force constants (Table 1). Considering that the densities are very similar for ZnO and GaN, bond stiffness is thus the primary reason for the observed differences in speed of sound. In Fig. 3, we plot detailed group velocities for each phonon mode in the first Brillioun zone as a function of frequency. From the figure, the difference in phonon group velocities is evident, and the differences at the low frequency modes (long wavelength acoustic modes) generally shows a factor of 1.4, which agrees well with the difference in speed of sound. Because of its quadratic dependence, such a factor should contribute a ~2 fold difference in thermal conductivity. Thus, it can be concluded that the difference in inter-atomic bond stiffness (and thus group velocity) is not enough to explain the eight-fold difference in observed thermal conductivity.

**Figure 4:**
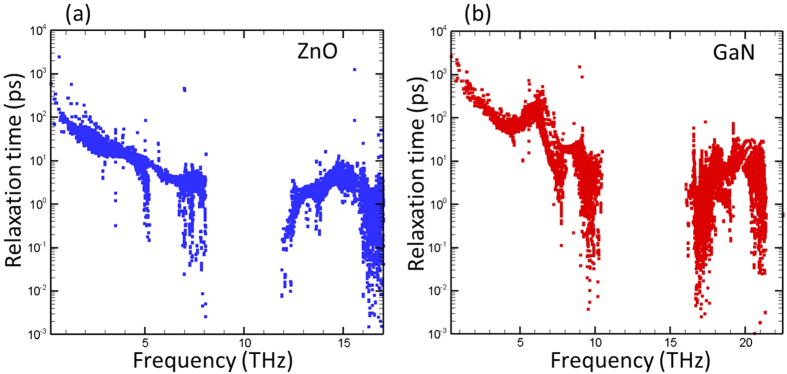
Phonon relaxation times as a function of frequency for ZnO and GaN calculated under SMRTA.

The third characteristic of phonons that can influence thermal conductivity is the phonon relaxation time, *τ*. Figure 4 shows *τ* values of ZnO and GaN as a function of frequency for all phonon modes. Strictly speaking, this calculation is only meaningful under the single mode relaxation time approximation (SMRTA) which is shown to under-predict the thermal conductivity since it treats both Normal and Umklapp scattering as resistive processes^34^. However, we found that SMRTA produce results within 4.5% of the iterative solution results for both ZnO and GaN at room temperature. From Fig. 4, it can be clearly seen that the *τ* of ZnO phonons are much smaller than those of GaN phonons. In the following sections, we further study the reason for the observed difference in relaxation times.

### Three phonon scattering phase space

**Figure 5:**
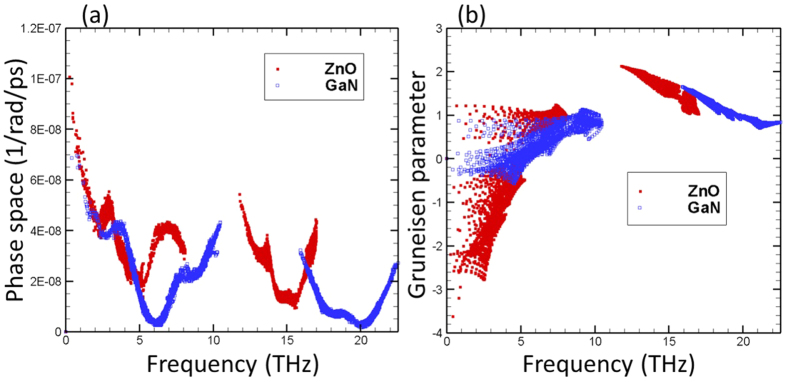
(**a**) Three phonon scattering phase space; and (**b**) Gruneisen parameter of ZnO and GaN as a function of frequency.

As seen in Fig. 2, one notable feature in the phonon dispersion is the gap between the highest acoustic phonon frequency and the lowest optical phonon frequency. It has been shown that such a gap can greatly hinder the three-phonon scattering since it makes satisfying the scattering rules (energy and quasi-momentum conservation) difficult, thus leading to longer phonon lifetimes^13^. It has been found that a larger gap often leads to higher thermal conductivity^22^,^35^. One of the major reasons for the high GaN thermal conductivity is associated with having such a large gap^13^. From the dispersion relation in Fig. 2, such frequency gap is much smaller for ZnO than that of GaN. To quantify the difference, we compare the three phonon scattering phase space for both wurtzite crystals, which roughly indicates the likelihood of phonons being scattered. The phase space for each mode is calculated as:^35^


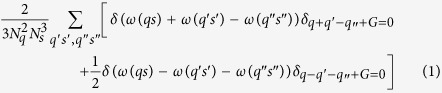


where *q*, *s* and *ω* refer to the wave-vector, polarization and angular frequency of a phonon mode, respectively. *N*_*q*_ refers to the total number of *q* points in the discretized first Brillioun zone in the calculation, while *N*_*s*_ is the total number of phonon branches. *G* is the reciprocal lattice vector. The prime and double prime superscripts are used to label different phonon modes involved in three-phonon scattering. From Fig. 5a, it is seen that the phase space of ZnO is larger than that of the GaN as expected from the smaller phonon frequency gap in ZnO dispersion. This tells us that phonons in ZnO will have more channels for scattering, resulting in relatively lower phonon relaxation times.

### Gruneisen parameter

The factor that determines the strength of each scattering channel is phonon anharmonicity. The anharmonicity of a crystal is usually characterized by the Gruneisen parameter, which describes how much phonon frequency gets shifted with changes in volume. We calculated the Gruneisen parameter for ZnO and GaN according to Eq. 2 for each phonon mode, and we compared some of them to available experimental data from Raman spectroscopy^36^. The equation of Gruneisen parameter is defined as:


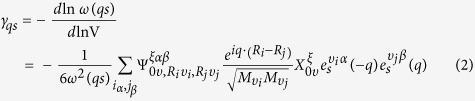


where *α*,*β*,*γ* represent the *x, y, z* component of Cartesian coordinates, respectively.*X* denotes the equilibrium atomic position, and 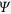 is the force constant. 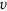 indicates the type of atom, and *R* is the translation vector between a specific unit cell and the primitive cell in the supercell. *i, j* refer to indices of atoms over all neighbors of atom 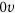. It is seen that our calculation agree well with the available experimental data (Table 2), which is an indication of the accuracy of the predicted cubic force constants from DFT calculations.

We further compare the modal Gruneisen parameter as a function of frequency for ZnO and GaN (Fig. 5b) and observe that the Gruineisen parameters of ZnO are generally larger than GaN, indicating that ZnO is more anharmonic. Hence, we conclude that the small phonon relaxation times of ZnO are due to the combined effect from the larger scattering phase space and higher anharmonicity.

### Mode Contribution to Thermal Conductivity

**Figure 6:**
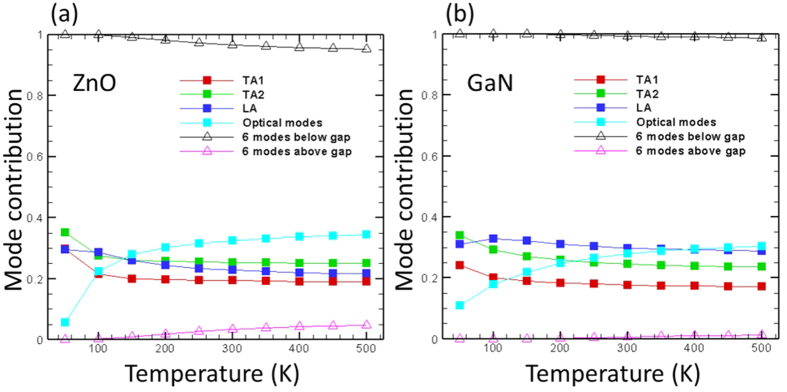
Mode contributions to total thermal conductivity of (**a**) ZnO and (**b**) GaN. The solid squared symbols are for thermal conductivity decomposition to three acoustic branches (TA1 and TA2: two transverse acoustic branches; LA: the longitudinal acoustic branch) and the sum of the optical modes. The open triangles are for decomposition to phonon modes below and above the frequency gap as seen in Fig. 2.

**Figure 7:**
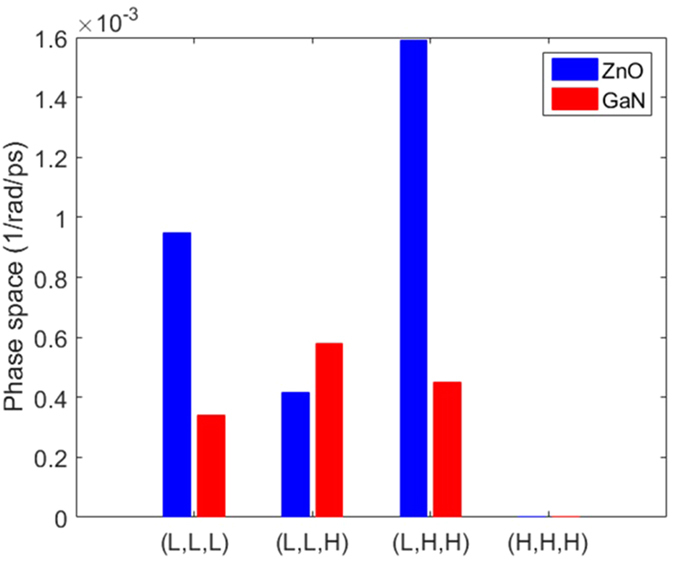
Three phonon scattering phase space divided into contributions from different scattering groups. “L” and “H” refer to phonon modes below and above the frequency gap, respectively.

We also calculate the relative contributions of acoustic and optical phonon modes in order to gauge their importance towards thermal conductivity (solid squares in Fig. 6). ZnO and GaN share a lot of similarities from the mode contribution perspective: acoustic modes dominate thermal conductivity at all temperatures, but optical modes become increasingly important with increase in temperature. We primarily attribute this to the increase in heat capacity of the optical modes as temperature increases. When decomposing thermal conductivity contributions from modes above and below the phonon frequency gap, it is seen that the modes below the frequency gap almost completely dominate thermal conductivity while those above the gap are not as important. Although their contributions to thermal conductivity are small, the modes above the frequency gap are not negligible since they participate in phonon scattering processes with other phonons. Figure 7 shows the contribution of different scattering processes towards the total phase space. The processes are divided into contributions from different three-phonon groups: (L,L,L), (L,L,H), (L,H,H), and (H,H,H), where “L” and “H” refer to phonon modes below and above the frequency gap, respectively. It is seen that for both ZnO and GaN, modes above the frequency gap are required for two types of dominant scattering processes (*i.e.*, (L,L,H) and (L,H,H)). The scattering processes involving two “H” modes are much more significant in ZnO than in GaN, which is due to the smaller frequency gap in the ZnO phonon dispersion. The smaller frequency gap will allow more three phonon groups (two “H” and one “L” modes) to satisfy the scattering rules (energy and quasi-momentum conservation) and thus lead to larger phase space and lower thermal conductivity.

### Nanoscale Size Effect

**Figure 8:**
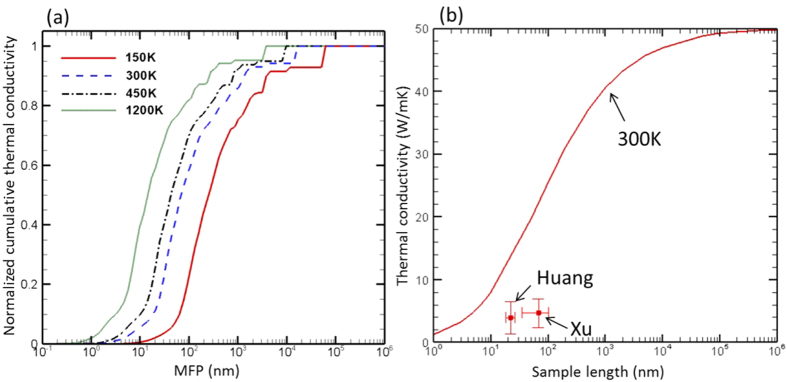
(**a**) Thermal conductivity accumulation as a function of phonon MFP at different temperatures; (**b**) Size dependent thermal conductivity of ZnO at 300 K and experimental data (Huang − Ref. 38 and Xu − Ref. 39).

In many devices, the ZnO layers are often grown on substrates such as a silicon wafer with native oxide. The lattice mismatch usually leads to large strain and thus grows crystals with small grain sizes. It is known that grain boundaries will scatter phonons, and thus the size of the crystalline domain acts as a limiting length for phonons MFP. It is thus worth studying this size effect on the thermal conductivity of ZnO. In Fig. 8a, we plot the thermal conductivity accumulation as a function of MFP, which offers insights on how size can influence thermal conductivity. It can be seen that size effect will be most pronounced in the range from 10–1000 nm. The curves shift to higher MFP when the temperature decreases, because phonons can have larger MFP at low temperatures where anharmonic phonon scattering becomes weak.

We further calculate the size dependent thermal conductivity at room temperature by using Matthiessen’s rule^37^ (Eq. 3) to combine the scattering due to anharmonicity and size effect (Fig. 8b).


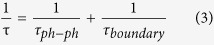


where *τ* is the effective phonon relaxation time, while *τ*_*ph-ph*_ and *τ*_*boundary*_ are those due to intrinsic anharmonic scattering and boundary scattering, respectively. It is found that a size of 100 nm can lead to a reduction of ~50% in thermal conductivity at room temperature with respect to bulk crystal. Huang *et al.*^38^ used transient thermoreflectance to measure the thermal conductivity of polycrystalline ZnO thin films deposited by a sol-gel method on a silicon substrate and found the values ranging from 1.4–6.5 W/mK. The grain sizes measured in these samples range from 18–26 nm. Xu *et al.*^39^ produced ZnO thin films with grain sizes ranging from 35–100 nm using reactive sputtering with different oxygen content, and the measured thermal conductivities (2.3–7.1 W/mK) are on a similar range as those from Huang *et al.*^38^. These data are plotted in Fig. 8b to compare with our calculations, but our predictions are notably larger than the experimental values. The discrepancy could be from other defects in the sample (*e.g.*, chemical residue, foreign particles, crystallinity, voids and so on) and perhaps as well as the uncertainties in the measurements. It should be worth performing systematic phonon spectroscopy^40^,^41^,^42^ to measure size dependent thermal conductivity of single ZnO crystals and compare to our calculations.

## Conclusions

Using first-principles lattice dynamics, we calculate the thermal conductivity of *w*-ZnO. The results (50 W/mK at room temperature) agree well with experimental data from laser flash measurements. However, this thermal conductivity is much lower than those measured from SThM, which is attributed to the possible uncertainty involved in samples and SThM measurements. We also compared ZnO thermal conductivity with that of GaN – another wide bandgap semiconductor very similar to ZnO. The ZnO thermal conductivity is found to be much smaller than GaN (50 vs. 400 W/mK at room temperature). Our analysis of phonon properties show such a difference roots from the combination of larger three-phonon scattering phase space (inferred by the smaller frequency gap in phonon dispersion), larger anharmonicity (Gruenisen parameter), and smaller phonon group velocities (weaker interatomic bonds) of ZnO as compared to GaN. The results from this work provide insight in converging in the large discrepancy in ZnO thermal conductivity in reported literature and provide practically useful information (*e.g.*, size effect, anisotropy and etc.) towards device design for ZnO-based electronics.

## Methods

The potential energy (*V*) and force (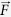) of a group of interacting atoms can be expanded using a Taylor series expansion with respect to the atomic displacement (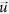) when the atoms vibrate around their equilibrium positions (Eqs (4 and 5), respectively).


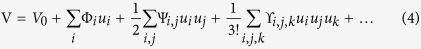



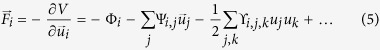


where 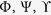 are the first-, second- and third-order force constants, respectively, while the subscripts represent atom indices. The second- and third-order force constants are also referred to as harmonic and cubic force constants, respectively. We only considered the force constants up to the third order in our calculations since higher order anharmonicity has limited effect on phonon scattering unless at very high temperatures^43^. The harmonic force constants are calculated using density functional perturbation theory (DFPT)^44^. Non-analytical terms due to the Columbic forces are added to the dynamical matrices^25^ with Born charges and the dielectric constant calculated from DFPT^44^. The cubic force constants are calculated using finite difference according to Eq. (5), where atoms are moved systematically by 0.01 Å and the resultant forces are calculated using density functional theory. The cubic force constants matrices are then constructed from this force-displacement data using the Thirdorder Python tool^45^. The phonon relaxation times and thermal conductivities are calculated using Fermi’s Golden rule^46^ with the iterative solution of the Boltzmann transport equation as implemented in our in-house code. We have also used the open source ShengBTE code^45^,^47^,^48^ to validate our calculations, and the same results were obtained.

All first-principles calculations are performed using Quantum-Espresso^49^. For both ZnO and GaN, norm-conversing pseudopotentials with Perdew-Zunger local density approximation (LDA)^50^ are used with a planewave cut-off of 50 Rydberg. Besides LDA, we have also tested the generalized gradient approximation (GGA) and Becke-Lee-Yang-Parr (BLYP) functionals, and they yielded thermal conductivity values within 7% of the LDA results. A Monkhorst-Pack^51^ mesh of 8 × 8 × 8 in the *q*-space is chosen based on a convergence test of the lattice energy. The lattice constants of the hexagonal primitive cell for ZnO were *a* = 3.187 Å, *c* = 5.152 Å, and those for GaN were *a* = 3.086 Å, *c* = 5.023 Å. The lattice constants for ZnO were optimized to remove any strain in the cell, and those of GaN were chosen so that the predicted phonon dispersion matches experimental results. It is worth noting that the optimized ZnO lattice constants yield phonon dispersion that agrees very well with experimental data (Fig. 1a). Fine tuning GaN lattice constants was necessary to match the experimental dispersion relation, both LDA and GGA^52^,^53^ did not produce phonon dispersion matching experiments when the lattice constants were relaxed to release the strain in the cell. It has been proven that such tuning of lattice constants does not lead to an error as the predicted thermal conductivity agrees exactly with those from ref. 13. The harmonic force constants are calculated with a *q*-space grid of 4 × 4 × 4, making the effective cutoff of the harmonic force constants larger than 9 Å. The calculations for cubic force constants were performed on a 3 × 3 × 3 supercell, and the cutoff range is set to the third nearest neighboring shell. No changes were found on the thermal conductivity when a larger supercell or longer cutoff ranges were used. The *q*-space grid of 20 × 20 × 20 was used for the Fermi’s Golden rule calculation, as this grid size was found to offer well-converged thermal conductivity values for both GaN and ZnO.

## Additional Information

**How to cite this article**: Wu, X. *et al.* Thermal Conductivity of Wurtzite Zinc-Oxide from First-Principles Lattice Dynamics - a Comparative Study with Gallium Nitride. *Sci. Rep.* **6**, 22504; doi: 10.1038/srep22504 (2016).

## Figures and Tables

**Table 1:** Comparison of physical properties between *w*-ZnO and *w*-GaN.

Properties	*w*-ZnO	*w*-GaN
Lattice type	wurtzite	wurtzite
Lattice constants (Å)	*a* = 3.25; *c* = 5.21^58^	*a* = 3.19; *c* = 5.19^59^
Atomic mass (a.m.u.)	65.4 (Zn); 16.0 (O)	69.7 (Ga); 14.0 (N)
Density (g/cm^3^)	5.61	6.15
Volumetric heat capacity (kJ/m^3^K)	2900	2800
Average speed of sound (m/s)	2760 (transverse); 6090 (longitudinal)^60^	4140 (transverse); 7885 (longitudinal)^61^
Largest 2^nd^ order constants (eV/Å^2^)	12.15	17.50
Elastic constants (GPa)	*E*_11_ = 210; *E*_12_ = 121; *E*_13_ = 105; *E*_33_ = 211; *E*_44_ = 43^60^	*E*_11_ = 390; *E*_12_ = 145; *E*_13_ = 106; *E*_33_ = 398; *E*_44_ = 105^62^
Phonon frequency gap (THz)	3.98	6.11

(Note: Data not from a reference are calculated in the present work).

**Table 2:** Gruneisen parameter of different modes at Brillouin Zone center.

Modes	Frequency (cm^−1^)	this work	Experiment^a^
E_1_ (LO)	557	1.5	1.4
E_2_ high	445	2.0	2.0
E_1_ (TO)	411	2.1	1.8
A_1_ (TO)	393	2.1	2.1
E_2_ low	89	−2.3	−1.6

^a^Ref. 36.
